# Control Work

**Published:** 1896-01

**Authors:** 


### CONTROL WORK.

*District of Columbia.* The health office of the District, through its veterinarian, is now engaged in enforcing the law regulating dairy-inspection. Under this law all milch-cows that supply milk to the city are inspected, and a certificate granted when found free from disease.

*New Jersey.* Another one of New Jersey's cities has erected a slaughtering establishment for the preparation of horseflesh as a food-product. The local board of health has reason to believe that diseased animals are destroyed, and an investigation is being pushed to determine the facts in the case.

A number of cattle that have been under the care of Veterinarian Conrow, of Moorestown, N. J., covering a period of two years, and which were tuberculous, as indicated by tuberculin-test, and had received at stated periods injections of tuberculin, were recently destroyed and many evidences of curative results were found present. The most of the tuberculous deposits were encysted and had undergone retrograde changes, indicating a termination of the power of the active bacilli.

*New York.* At Malone a herd of thirty high-bred cattle were examined with tuberculin by the State Board of Health. Thirty proved tuberculous, and were ordered killed. Other herds in the same county will be examined at once.

Fifty-nine cattle afflicted with tuberculosis were killed at Deposit recently by the New York State Board of Health.

